# A novel model of the continual reassessment method in Phase I trial

**DOI:** 10.1038/s41598-023-28148-4

**Published:** 2023-03-28

**Authors:** Weijia Zhang, Wanni Lei, Xiaojun Zhu

**Affiliations:** 1 Center of Statistical Research and School of Statistics, Southwestern University of Finance and Economics, Chengdu, 611130 Sichuan China; 2 Department of Applied Mathematics, Xi’an Jiaotong-Liverpool University, Suzhou, 215123 Jiangsu China; 3 Department of Financial and Actuarial Mathematics, Xi’an Jiaotong-Liverpool University, suzhou, 215123 Jiangsu China

**Keywords:** Phase I trials, Statistics

## Abstract

For the model-based designs, the continual reassessment method (CRM) is widely used to identify the maximum tolerated dose (MTD) in phase I clinical trials. To improve the performance of classic CRM models, we propose a new CRM and its dose-toxicity probability function based on the Cox model whatever the treatment response is immediately observed or delayed. In the process of dose-finding trial, we can use our model in situations when either the response is delayed or not and can derive the likelihood function and posterior mean toxicity probabilities to find the MTD. Simulation is carried out to evaluate the performance of the proposed model with the classic CRM models. We also evaluate the operating characteristics of the proposed model by the Efficiency, Accuracy, Reliability, and Safety (EARS) criteria.

## Introduction

Clinical trials are carried out on the human body with the aim of solving certain medical problems. Such particularity of clinical trials determines that they should not only be rigorous but also abide by ethics^1^. Clinical trials are often conducted and funded by the pharmaceutical company to justify whether a new drug is effective, and there are four phases in the clinical trials of producing new drugs. Phase I trial is the foundation of phase II, III and IV clinical trials, and it normally includes 20–30 people, aiming to identify the MTD, which is the most toxic dose referred to patients, so that if the dose higher than the MTD, patients can not tolerate and may cause the fatal death and if the dose lower than the MTD, patients would suffer ineffective dosage and may miss the best time for treatment. So the therapeutic effect of the drug can be maximized when the patients are treated at the MTD, and then the MTD or dose level less than the MTD is recommended to phase II. Finding the MTD accurately and efficiently is very crucial in phase I which is also called the dose finding trial. The MTD is defined as the dose whose toxicity probability is closest to a target toxicity probability, usually say 33%^2^. In the process of dose-finding trial, the dose assigned to the next patient is corresponding to responses of former patients^3^. Many methods are applied to determine the MTD, including algorithm-based, model-assisted, and model-based designs.

Algorithm-based designs include the 3+3 design^4^ and its extensions, the accelerated titration design^5^, and the biased coin design^6^. Model-assisted designs include the bayesian optimal interval design^7^, the modified toxicity probability interval (mTPI) design^8^, and the keyboard design^9^. Model-based designs require some statistical background to model the relationship between dose and toxicity probability, including the CRM^10^, the dose escalation with overdose control^11^, and the Bayesian logistic regression model^12^. The key point of CRM method is to assume a parametric function between dose and toxicity probability, and based on the dose-toxicity curve, we can estimate the true toxicity probability by updating the observed data from each treated patient. The dose with the updated toxicity probability closest to the target rate will be assigned to the next cohort of patients.

In the literature, there are many works on modifications of CRM models. The issue of overdosing is considered by Faries^13^, Goodman *et al*.^14^, and Ishizuka and Ohashi^15^. Piantadosi *et al*.^16^ propose a modified CRM for cytotoxic drugs to fit the limited sample size of the phase I trials. Lee and Cheung^17^ and Cheung^18^ investigate the model calibration for the CRM with single skeleton. Yin and Yuan ^19^ propose the Bayesian model averaging continual reassessment method (BMA-CRM) and suggest to use multiple skeletons when conducting the CRM. A natural problem followed by the present of BMA-CRM is that how to specify multiple skeletons. To overcome this problem, Pan and Yuan^20^ define the equivalence of multiple skeletons and propose a method to choose skeletons for BMA-CRM models.

To incorporate grade information, Yuan *et al*.^21^ further modify the CRM by the quasi-Bernoulli likelihood, and Chia-Wei Hsu *et al*^22^ provide a R package named UnifiedDoseFinding for non-binary outcomes dose-finding trials. Since practical resources are scarce to conduct the CRM, Wheeler *et al*.^23^ present some comprehensive recommendations and guidelines to support clinicians to implement and report dose-finding trials using the CRM. Wages and Petroni^24^ develop a web tool for designing and conducting the CRM. Recently, North *et al*.^25^ extend the CRM and present a novel two-stage dose assignment procedure which combines the rule-based design with the CRM. Finally, Liu *et al*.^26^ develop a bridging CRM to find the MTD in different ethnic populations.

Substantial efforts have been made on the modification of the CRM, however, only few of these modified CRMs consider the late-onset toxicity . When the phase I trials are conducted, we have to specify a certain observation window (normally 28 days) for dose-limiting toxicity (DLT) assessment. In general, the basic method is that we need to classify the toxicity after treatment whether is immediately observed in the window or delayed out of the window $\tau$. If the observation is censored at time $\mu _i$, we still calculate the conditional probability of the occurrence toxicity within window, i.e., $Pr(t_i<\tau |t_i>\mu _i)$ ^27^. In the paper, we aim to develop a new CRM to deal with the treated patient if experiences DLT in or out of the observation window simultaneously. This goal motivates us to develop a model which can incorporate both the basic binary and the delayed responses. Ideally, we can only observe once in the observation window and no delayed toxicity out of the window. But in fact, the toxicity may be delayed and we ignore delayed responses in the trial using classic CRMs. Our idea is that the model can deal with immediately toxic or late onset toxic response since our model can include all the information of treatment effects. To save time and human cost, we only observe once in the evaluation window, nevertheless we cannot observe delayed response, our model still works well. We develop the Cox model to modify the classic CRMs. The information of delayed response and the initial guess of toxicity probability can be included in the Cox model, which is convenient for the future study of mixture of immediate and non-immediate responses. As we know, the Cox model is commonly used in survival analysis, and its technique may be an important approach in clinical research if we collect the real data^28^. The transformation of Cox model in clinical trial is used to analyze the patients’ responses if censored or not , what is more, we can not distinguish the delayed response with the normal ones. Simulations by R program show that the proposed new model has desirable operating characteristics and enhances the performance of the CRM.

The rest of this paper is organized as follows, in “Methods” section, we first derive the new toxicity probability function by the Cox model, and then provide the detailed dose-finding algorithm for our new model. In “Simulation studies” section, we carry out a detailed R simulations to compare and evaluate the performance of our new model with the classic CRM models with three pre-specified dose-toxicity curves. Finally, we end with conclusions and a brief discussion.

## Method

### Modified CRM model based on the Cox model

Let $( p_1,\dots , p_J )$ be the skeleton of the *J* doses, $p_1< \dots < p_J$, and let $\pi (t, p_i)$ denote the probability that patients experience DLT within a period t. We define the “toxic response” in the dose finding trial as the event in survival analysis. Further, we can use the Cox model to derive the explicit formula for $\pi (t, p_i)$. Hence, the Cox model introduces a new approach of the CRM model, and the explicit formula for the hazard rate function $h(t, p_i)$ is as follows,

1 $$\begin{aligned} h(t,p_i)=h_{0}(t) \exp \left( \alpha p_i\right) =\frac{f(t, p_i)}{S(t, p_i)} \quad \end{aligned}$$

where $\alpha$ is the unknown parameter, $f(t, p_{i})=\pi ^{\prime }(t, p_i)$, and $S(t, p_i)=P(T> t | p_i)$. Suppose all the patients only been observed within *t*, and we regard these patients having a toxic response after *t* as no response. From equation (1), we can derive $S(t)=e^{-\int _{0}^{t} h(u) d u}$, and obtain the explicit expression for $\pi ( t,p_{i})$ as:

2 $$\begin{aligned} \pi (t,p_{i})= 1-e^{-\int _{0}^{t} h_{0}(u) \exp \left( \alpha p_{i}\right) d u} \quad \end{aligned}$$

Further, let $\int _{0}^{ t} h_{0}(u) du$= *C*, then the probability of a patient can be observed a toxic response within *t* at dose *i* is:

3 $$\begin{aligned} \pi (i)= 1-e^{-exp(\alpha p_{i})C} \quad \end{aligned}$$

where C is a constant, and $\alpha$ is assigned to have the prior distribution $f(\alpha )$.

Let $D=\left\{ \left( n_{i}, y_{i}\right) , i=1, \ldots , J\right\}$ be the observed data, where $n_i$ is the number of patients who have been assigned to dose *i*, and $y_i$ is the number of DLTs observed at dose *i*. Then the likelihood function is:

4 $$\begin{aligned} L(\alpha \mid D)=\prod _{i=1}^{J}\left\{ 1-e^{-\exp \left( \alpha p_{i}\right) C}\right\} ^{y_{i}}\left\{ 1-\left[ 1-e^{-\exp \left( \alpha p_{i}\right) C}\right] \right\} ^{n_{i} - y_{i}} \end{aligned}$$

Now the posterior mean toxicity probability at dose *i* is:

5 $$\begin{aligned} {\hat{\pi }}_{i}=\int \left( 1-e^{-\exp \left( \alpha p_{i}\right) C}\right) \frac{L(D \mid \alpha ) f(\alpha )}{\int L(D \mid \alpha ) f(\alpha ) \textrm{d} \alpha } \textrm{d} \alpha , \quad i=1, \cdots , J \end{aligned}$$

Similar to the classic CRM designs, we update the posterior mean toxicity probability through the successively entering of patients, which guides the dose transition in the trial. The procedure will not be terminated until the last cohort of the patients. The dose level with final update of the posterior mean toxicity probability closest to the target should be chosen as the MTD.

### Dose-finding algorithm

To conduct the modified CRM model, we take the following steps:

- Step 1: Choose a prior $f(\alpha )$ distribution for $\alpha$.
- Step 2: The first cohort of patients is assigned to the lowest dose, and $D=\left\{ \left( n_{i}, y_{i}\right) , i=1, \ldots , J\right\}$.
  6 $$\begin{aligned} Likelihood \quad function: L(D \mid \alpha )=\prod _{i=1}^{J}\left\{ 1-e^{-\exp \left( \alpha p_{i}\right) C}\right\} ^{y_{i}}\left\{ e^{-\exp \left( \alpha p_{i}\right) C}\right\} ^{n_{i}- y_{i}} \end{aligned}$$
- Step 3: Use previous information and the Bayes’ formula, we can estimate the updated toxicity probability ${\hat{\pi }}_{i}$ of each dose.
  7 $$\begin{aligned} {\hat{\pi }}_{i}=\int (1-e^{-\exp \left( \alpha p_{i}\right) C}) \frac{L(D \mid \alpha ) f(\alpha )}{\int L(D \mid \alpha ) f(\alpha ) \textrm{d} \alpha } \textrm{d} \alpha , \quad i=1, \cdots , J \end{aligned}$$
- Step 4: The selection of dose for next cohort of patients depends on the dose $i^{*}$, where $i^{*}=\underset{i \in (1, \cdots , J)}{{\text {argmin}}}\left| {\hat{\pi }}_{i}-p_{\textrm{T}}\right|$, where $p_{\textrm{T}}$ is the physician-specified toxicity target.
  - If $d_i^{*}$ $>d_i$, we escalate the dose level from *i* to $i + 1$.
  - If $d_i^{*}$ $<d_i$, we deescalate the dose level from *i* to $i - 1$.
  - If $d_i^{*}= d_i$, we stay at the current dose level *i*.
- Step 5: Repeat steps 1-4 until all the patients enter the trial. The dose with the posterior mean toxicity probability closest to the target is defined as the MTD.

## Simulation studies

### Simulation setting

In the simulation, the prior distribution is taken as a normal distribution with mean 0 and deviation $\sigma =2$ ^19^. We simulate each trial for 2000 times by R to get more accurate results. We also compared the performance of the new model with the power model $\pi (p_{i}) = p_{i}^{\exp (\alpha )}$, logistic model $\pi (p_{i}) =\frac{\exp (-2+\alpha p_{i})}{1+\exp (-2+\alpha p_{i})}$ and hyperbolic tangent model $\pi (p_{i}) = [(\tanh (p_{i})+1) / 2]^{\alpha } = \left[ \frac{\exp (p_{i})}{\exp (p_{i})+\exp (-p_{i})}\right] ^{\alpha }$ used in the classic CRM designs under EARS criteria proposed by Zhang *et al*. ^29^.

We simulate six different toxicity scenarios with eight different dose levels in each trial, these scenarios are shown in Table 1. The target toxicity probability is 30%. The true toxicity probability in each scenario is assumed to be monotonically increasing with the dose level. In each scenario, we list the true toxicity probability in the first row, and the corresponding skeleton in the second row, which is generated from the getprior() function in R. The MTDs of the six different scenarios are marked in bolditalic. These different true toxicity probabilities present some different situations in practice. To be more specific, in scenario 1, the toxicity probability of each dose locates on a relatively low level, only the last dose reaches to 30%. In scenarios 2, 3, 4, the toxicity probabilities increase slightly at the lower doses, however, they increase sharply when at higher dose levels. In scenarios 5 and 6, the toxicity probabilities are relatively high at lower dose levels, for instance, toxicity probability is 0.3 at dose 3 in scenario 5, and 0.2 at dose 1 in scenario 6. In these two cases, over half of the doses are overly toxic, i.e., the patient treated with the dose whose toxicity probability is higher than 30%.

We confirm that all methods were performed in accordance with the relevant guidelines and regulations.

**Table 1:** Six Scenarios with different MTDs.

Scenario	Target$=30\%$	Dose1	Dose2	Dose3	Dose4	Dose5	Dose6	Dose7	Dose8
Scenario1	True toxicity rate	0.02	0.03	0.05	0.06	0.07	0.09	0.10	**0.30**
	Skeleton	0.0002	0.002	0.008	0.026	0.06	0.12	0.20	0.30
Scenario2	True toxicity rate	0.02	0.03	0.04	0.06	0.08	0.10	**0.30**	0.50
	Skeleton	0.002	0.008	0.026	0.06	0.12	0.20	0.30	0.40
Scenario3	True toxicity rate	0.03	0.07	0.10	0.15	0.20	**0.30**	0.50	0.70
	Skeleton	0.008	0.026	0.06	0.12	0.20	0.30	0.40	0.50
Scenario4	True toxicity rate	0.02	0.03	0.05	0.07	**0.30**	0.50	0.70	0.80
	Skeleton	0.03	0.06	0.12	0.20	0.30	0.40	0.50	0.59
Scenario5	True toxicity rate	0.06	0.15	**0.30**	0.55	0.60	0.65	0.68	0.70
	Skeleton	0.20	0.30	0.40	0.50	0.60	0.65	0.70	0.75
Scenario6	True toxicity rate	0.20	**0.30**	0.40	0.50	0.60	0.65	0.70	0.75
	Skeleton	0.30	0.39	0.48	0.57	0.64	0.71	0.76	0.81

True toxicity rate at MTDs are in [bold].

**Table 2:** Probability of each dose being chosen as the final MTD according to different models.

Model	Dose1	Dose2	Dose3	Dose4	Dose5	Dose6	Dose7	Dose8
Scenario1								
Power model	0	0	0	0	0	0	0	1
Logistic model	0	0	0	0	0	0	0.0125	0.9875
Hyperbolic tangent model	0	0	0	0	0	0.0030	0.1245	0.8725
New model	0	0	0	0	0	0	0.0045	**0**.**9955**
Scenario2								
Power model	0	0	0	0	0	0	0.5015	0.4985
Logistic model	0	0	0	0	0	0.0460	0.6455	0.3085
Hyperbolic tangent model	0	0	0	0	0.0065	0.1695	0.6025	0.2215
New model	0	0	0	0	0	0.0040	**0**.**6280**	0.3680
Scenario3								
Power model	0	0	0	0	0	0.5700	0.4285	0.0015
Logistic model	0	0	0	0	0.0895	0.7290	0.1755	0.0060
Hyperbolic tangent model	0	0.0015	0.0065	0.0535	0.2565	0.5130	0.1615	0.0075
New model	0	0	0	0	0.0070	**0.7400**	0.2485	0.0045
Scenario4								
Power model	0	0	0	0	0.5120	0.4850	0.0030	0
Logistic model	0	0	0	0.0630	0.7500	0.1815	0.0055	0
Hyperbolic tangent model	0	0	0.0005	0.1505	0.6490	0.1980	0.0020	0
New model	0	0	0	0.0050	**0**.**7230**	0.2690	0.0030	0
Scenario5								
Power model	0	0.0310	0.8685	0.0980	0.0025	0	0	0
Logistic model	0	0.2170	0.6575	0.1200	0.0035	0.0015	0.0005	0
Hyperbolic tangent model	0.006	0.2390	0.6130	0.1415	0.0005	0	0	0
New model	0	0.1055	**0**.**7535**	0.1335	0.0070	0.0005	0	0
Scenario6								
Power model	0.0200	0.6800	0.2495	0.0450	0.0050	0.0005	0	0
Logistic model	0.2090	0.4850	0.2515	0.0425	0.0105	0.0005	0.0010	0
Hyperbolic tangent model	0.3810	0.4220	0.1740	0.0215	0.0015	0	0	0
New model	0.0740	**0**.**5615**	0.2850	0.0680	0.0100	0.0010	0	0.0005

Results of new model at MTDs are in [bold].

### Results evaluation

#### Final selected MTD

We study the classic CRMs and our model to compare their percentages of the final MTD selection. The higher the percentage, the more patients assigned to the dose. Table 2 shows the percentage of the final chosen MTD in six scenarios. Each row of the table is the percentages of patients assigned to each dose during the trial. For example, in scenario 3, the MTD is at dose 6. The fourth row indicates that 0.7%, 74%, 24.85% and 0.45% of the patients are assigned to the dose 5, 6, 7 and 8, respectively. That is our model allocates most of the patients to the MTD with dose 6.  The simulation results of our new model at MTDs are shown in bold.

In scenario 1, the MTD is located at the highest dose level, i.e., dose 8, and all the four CRM models perform well. All the four models select the right MTD with a percentage higher than 87%. The power model has the best result in this scenario. It is true that the new model is the second-best in this scenario with 99.55%, however, this number is almost as good as the best result 100%. In scenario 2, dose 7 is the MTD, and the logistic model has the best result. Again, the new model is the second best with only 1.75% lower than the best result of 64.55%. In particular, in scenario 3 our new model becomes the best compared with the other three models. It has a percentage of 74% to choose the right MTD. Nevertheless, this number is significantly larger than the worst-case with 51.3%. Similarly, in scenarios 4, 5, and 6, the new model selects the right MTD with the second-best results, just a little bit lower than the best results. In scenarios 4 and 5, the accuracy of our new model is higher than 72%, indicating that our new model is competitively reasonable. Particularly, in scenario 2, 30.85% and 36.80% of patients choose the dose 8 as the MTD in logistic and our models, respectively. In all other scenarios have very similar behaviors. This does not necessarily lead to over toxic dose selection of the MTD at the higher doses, because the logistic model tends to overestimate the toxicity probabilities due to ignoring or discarding the missing data of delayed responses, which results in dose escalation tends to be less aggressive. On the other hand, our design underestimate the dose toxicity during the observation period and thus dose escalation tends to be more aggressive so that the value 36.8% is higher than that in the logistic model, but our design consider the late-onset data which are in fact available before we determine the MTD. As a result, dose escalation in our design is relatively conservative.

**Figure 1:**
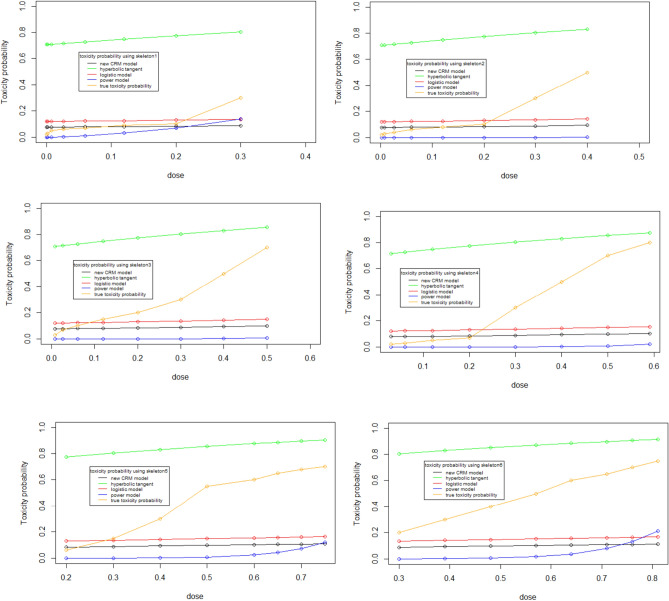
Toxicity probability using 6 different skeletons.

Figure 1 show the initial dose-toxicity curves of the new model and three classical CRM models using six different skeletons respectively. The parameter $\alpha$ in each model is set as 0.5. In general, the more the shape of initial curve is similar to the true dose-toxicity curve, the more correctly identify the MTD.

#### EARS

Table 3 shows the results of EARS in our simulation.

1. E reports for **Efficiency**, and E1 shows the percentage of simulations in which patients are treated with any dose lower than the MTD. E2 shows the mean and standard deviation of the percentage of patients treated with any dose lower than the MTD. In fact, these assignments lower than the MTD are ineffective. To design a good model, we expect the results as small as possible.
  Clearly, the proposed model is the second-best in all scenarios by the E criterion from the table. Therefore, we can conclude that this new model is efficient and performs well.
2. A represents for **Accuracy**, and A1 reports the percentage of simulations in which patients are assigned to the right MTD. A2 states the mean and standard deviation of the percentage of patients assigned to the MTD. A3 represents the percentage of simulations in which over half of patients are assigned to the right MTD. To design a good model, we expect the results of A1, A2, and A3 as large as enough.
  Table 4 shows that our proposed model has the best or the second-best performance in the most scenarios.
3. R stands for **Reliability**, and R1 is the percentage of simulations in which greater than half of the patients are assigned to any dose larger than the MTD. In this case, we measure the aspect of overly toxic. Similarly, R2 shows the percentage of simulations in which lower than 1/6 of the patients are assigned to the MTD. In this case, we measure the aspect of accuracy. A good design will has small results of R1 and R2.
  Although the results of the proposed model are not always the best or the second-best, the results are still small enough. Our new model outperforms and is reliable under the R criterion.
4. S expresses **Safety**, and S1 calculates the percentage of simulations in which patients are assigned to any dose higher than the MTD. S2 states the mean and standard deviation of the proportion of patients treated with any dose higher than the MTD. To design a good model, we expect the results of S1 and S2 to be small.
  Overall, it is true that the results of the proposed model under the S criterion are not as good as the previous results under the E and A criteria, but we still can conclude that our new model is safe.

**Table 3:** EARS for the power, logistic, hyperbolic tangent model and the new model.

Model	Efficiency		Accuracy			Reliability		Safety	
Scenario1	E1	E2	A1	A2	A3	R1	R2	S1	S2
Power model	0	0.033(0)	1	0.767(0)	1	0	0	0	NaN(NaN)
Logistic model	0.013	0.034(0.010)	0.988	0.763(0.026)	0.997	0	0	0	NaN(NaN)
Hyperbolic tangent model	0.128	0.056(0.052)	0.873	0.610(0.162)	0.769	0	0.018	0	NaN(NaN)
New model	0.005	0.033(0.004)	0.996	0.766(0.011)	1	0	0	0	NaN(NaN)
Scenario2									
Power model	0	0.033(0)	0.502	0.200(0.202)	0.092	0.700	0.550	0.499	0.600 (0.202)
Logistic model	0.046	0.036(0.023)	0.646	0.312(0.198)	0.180	0.427	0.283	0.309	0.474(0.211)
Hyperbolic tangent model	0.176	0.065(0.056)	0.603	0.390(0.147)	0.202	0.105	0.073	0.222	0.218(0.196)
New model	0.004	0.034(0.011)	0.628	0.298(0.211)	0.191	0.470	0.341	0.368	0.499(0.214)
Scenario3									
Power model	0	0.033(0)	0.570	0.252(0.224)	0.146	0.555	0.453	0.430	0.291(0.156)
Logistic model	0.090	0.039(0.036)	0.729	0.420(0.180)	0.346	0.080	0.105	0.182	0.193(0.125)
Hyperbolic tangent model	0.318	0.096(0.083)	0.513	0.352(0.171)	0.183	0.038	0.147	0.169	0.083(0.119)
New model	0.007	0.034(0.013)	0.740	0.398(0.220)	0.381	0.127	0.199	0.253	0.216(0.138)
Scenario4									
Power model	0	0.033(0)	0.512	0.200(0.185)	0.073	0.654	0.522	0.488	0.222(0.109)
Logistic model	0.063	0.039(0.034)	0.750	0.444(0.185)	0.416	0.097	0.093	0.187	0.134(0.098)
Hyperbolic tangent model	0.151	0.085(0.067)	0.649	0.444(0.138)	0.328	0.056	0.030	0.200	0.073(0.099)
New model	0.005	0.034(0.016)	0.723	0.412(0.237)	0.422	0.176	0.216	0.272	0.150(0.115)
Scenario5									
Power model	0.031	0.040(0.040)	0.869	0.655(0.215)	0.769	0.075	0.035	0.101	0.053(0.084)
Logistic model	0.217	0.107(0.134)	0.658	0.481(0.168)	0.476	0.011	0.039	0.126	0.061(0.063)
Hyperbolic tangent model	0.245	0.230(0.141)	0.613	0.432(0.174)	0.356	0.013	0.079	0.142	0.022(0.060)
New model	0.106	0.077(0.126)	0.754	0.502(0.207)	0.553	0.028	0.085	0.141	0.069(0.073)
Scenario6									
Power model	0.020	0.040(0.045)	0.680	0.501(0.313)	0.523	0.267	0.223	0.300	0.077(0.116)
Logistic model	0.209	0.162(0.204)	0.485	0.374(0.206)	0.287	0.070	0.188	0.306	0.077(0.081)
Hyperbolic tangent model	0.381	0.603(0.281)	0.422	0.266(0.189)	0.110	0.031	0.326	0.197	0.022(0.071)
New model	0.074	0.094(0.161)	0.562	0.360(0.250)	0.336	0.160	0.297	0.365	0.091(0.096)

## Compared with TITE-CRM

We compare the final MTD selection with the power model (CRM), TITE-CRM^27^ and our proposed design. In simulation studies, we use the similar setting introduced by Yin *et al.*^27^. In all the CRM-type designs, the prespecified toxicity probabilities are 0.1, 0.2, 0.3, 0.4, 0.5, 0.6 for 6 dose levels, the target toxicity probability is 30% and 6 scenarios are listed in Table 4. We also include a scenario where all the available doses are toxic and a safety rule to stop early for the trial ( A safety rule is defined below). We present the percentage of trials which stop early in the “Early Stop” column. In Table 4, the first row of each scenario is the true toxicity probabilities. Row 2, 3, and 4 represent the final MTD selection of the CRM with power model, TITE-CRM, and the proposed new design. In scenario 1, the MTD is at the dose 4, our design performs best, and yields the highest percentage of MTD selection. In scenario 3, the MTD is at the last dose, our new design performs best, which is much better than the performance of the TITE-CRM. In scenario 4, the MTD is at the dose 4, our design performs best, and yields the highest percentage of MTD selection, and the TITE-CRM performs the worst. In scenarios 2 and 5, the percentages of the final MTD selection of our design are not the highest, yielding 52.5% and 60%, but are not much worse than the CRM and TITE-CRM. In scenario 6, all of the doses are very toxic, the CRM and TITE-CRM have the percentages of 24.2% and 25.7%, while our design can stop the trial early. As expected, in most cases, our design outperforms the CRM and TITE-CRM, and improves the selection of the MTD by approximately 7%, especially in scenario 3, our design improves the percentage by 47% over the TITE-CRM designs. Clearly, our design can resolve the delayed toxicity probability issue.

**Safety rule** Always treat the first patients with dose 1. If the average of the posterior probability of dose 1 and dose 2 given the response of the first patient is greater than the target $p_T$, then terminate the trial because the doses may be overly toxic. Otherwise, continue the trial until reaching the maximum sample size.

**Table 4:** Simulation study comparing the results of CRM, TITE-CRM and the new model.

Design	Recommendation percentage at dose level						Early Stop
	1	2	3	4	5	6	
Scenario 1	0.07	0.12	0.17	0.3	0.45	0.6	
CRM	0.000	0.020	0.235	**0**.**590**	0.152	0.003	0.000
TITE-CRM	0.000	0.020	0.238	**0**.**572**	0.165	0.005	0.000
New model	0.000	0.000	0.023	**0**.**650**	0.194	0.031	0.104
Scenario 2	0.04	0.08	0.12	0.15	0.3	0.5	
CRM	0.000	0.000	0.018	0.221	**0**.**646**	0.115	0.000
TITE-CRM	0.000	0.002	0.027	0.239	**0**.**614**	0.118	0.000
New model	0.000	0.000	0.000	0.236	**0**.**525**	0.233	0.007
Scenario 3	0.05	0.14	0.18	0.2	0.23	0.3	
CRM	0.000	0.014	0.082	0.178	0.321	**0**.**405**	0.000
TITE-CRM	0.000	0.017	0.116	0.233	0.340	**0**.**294**	0.000
New model	0.000	0.000	0.001	0.057	0.170	**0**.**764**	0.009
Scenario 4	0.08	0.1	0.2	0.3	0.4	0.55	
CRM	0.000	0.021	0.267	**0**.**500**	0.199	0.011	0.001
TITE-CRM	0.001	0.021	0.272	**0**.**496**	0.198	0.013	0.000
New model	0.000	0.000	0.019	**0**.**547**	0.267	0.072	0.096
Scenario 5	0.01	0.02	0.04	0.06	0.3	0.5	
CRM	0.000	0.000	0.003	0.111	**0**.**754**	0.132	0.000
TITE-CRM	0.000	0.000	0.005	0.115	**0**.**723**	0.157	0.000
New model	0.000	0.000	0.000	0.157	**0**.**600**	0.243	0.000
Scenario 6	0.5	0.6	0.7	0.8	0.85	0.9	
CRM	0.242	0.002	0.000	0.000	0.000	0.000	**0**.**756**
TITE-CRM	0.257	0.001	0.000	0.000	0.000	0.000	**0**.**742**
New model	0.000	0.000	0.000	0.000	0.000	0.000	**1**.**000**

Results at MTDs are in [bold].

## Conclusion

The main goal of phase I clinical trial is to correctly obtain the MTD as well as assign fewer patients to the overly toxic and ineffective dose levels. To achieve this goal and improve the performance of the classic CRM models, we propose a novel CRM design with a new dose-toxicity probability function. Results of the simulations exhibits that our proposed model is either the best or the second-best in all scenarios and is more robust. Then we use the EARS to evaluate the performance of the proposed model, and the results demonstrate that our new dose-finding method is more reliable, particularly we compare the performances of classic CRM, TITE-CRM and our design, and the simulation studies also show that most of the time, our design outperforms others. The contribution of our new model includes the information on both the toxicity and delayed responses of patients, but we do not need to classify the observation whether it is immediate or not. Involving the time, we can study some future works related to the delayed toxicity probability if we have real data. It is very challenge to deal with the missing data of the delayed responses in the real life due to the limited observation period. We often discard this information on the patients who did not have any toxic responses in the observation period but might experience DLTs beyond that period. But in this paper, our model automatically include this information and we can resolve this challenge part in the trial, and the simulation study looks good. However, the delayed toxic response may affect the accuracy of the MTD and will naturally be of interest to consider the delayed toxicity response when identifying the MTD. Our work on this problem is under progress and we hope to report our findings in the future.

## Data Availability

We generate the data using R and real data during the current study are not available for public due to some privacy of the patients, hospitals and pharmaceutical companies.
